# Exploring the Link between Photosystem II Assembly and Translation of the Chloroplast *psbA* mRNA

**DOI:** 10.3390/plants9020152

**Published:** 2020-01-25

**Authors:** Prakitchai Chotewutmontri, Rosalind Williams-Carrier, Alice Barkan

**Affiliations:** Institute of Molecular Biology, University of Oregon, Eugene, OR 97403, USA; pchotewu@uoregon.edu (P.C.); rozzz@uoregon.edu (R.W.-C.)

**Keywords:** photosystem II, *psbA*, translation, chloroplast, plastid, ribosome profiling, Arabidopsis, maize

## Abstract

Photosystem II (PSII) in chloroplasts and cyanobacteria contains approximately fifteen core proteins, which organize numerous pigments and prosthetic groups that mediate the light-driven water-splitting activity that drives oxygenic photosynthesis. The PSII reaction center protein D1 is subject to photodamage, whose repair requires degradation of damaged D1 and its replacement with nascent D1. Mechanisms that couple D1 synthesis with PSII assembly and repair are poorly understood. We address this question by using ribosome profiling to analyze the translation of chloroplast mRNAs in maize and Arabidopsis mutants with defects in PSII assembly. We found that OHP1, OHP2, and HCF244, which comprise a recently elucidated complex involved in PSII assembly and repair, are each required for the recruitment of ribosomes to *psbA* mRNA, which encodes D1. By contrast, HCF136, which acts upstream of the OHP1/OHP2/HCF244 complex during PSII assembly, does not have this effect. The fact that the OHP1/OHP2/HCF244 complex brings D1 into proximity with three proteins with dual roles in PSII assembly and *psbA* ribosome recruitment suggests that this complex is the hub of a translational autoregulatory mechanism that coordinates D1 synthesis with need for nascent D1 during PSII biogenesis and repair.

## 1. Introduction

Photosystem II (PSII) is a large protein–pigment complex whose light-driven water splitting activity is the foundation of oxygenic photosynthesis (reviewed in [1,2]). PSII localizes to thylakoid membranes of cyanobacteria and chloroplasts, where it consists of a highly conserved core complex surrounded by distinct light harvesting complexes. The PSII core contains approximately fifteen proteins in 1:1 stoichiometry, which serve as scaffolds to organize the numerous pigments and prosthetic groups that mediate light-absorption, charge separation and electron transport. This structure is assembled in an ordered pathway with the assistance of accessory factors that are, in many cases, conserved between cyanobacteria and chloroplasts [3]. Furthermore, PSII is a highly dynamic structure due to the damaging effects of light on its D1 reaction center protein. PSII repair involves an elaborate process that includes partial disassembly of the complex, proteolysis of damaged D1, new D1 synthesis, and re-assembly of the complex [4,5,6]. 

PSII biogenesis in plants and algae is further complicated by the fact that genes encoding PSII subunits are distributed between the nuclear and chloroplast genomes. During the biogenesis of chloroplasts, chloroplast-encoded PSII subunits are synthesized at rates that are roughly proportional to their stoichiometry in PSII [7,8]. However, in mature chloroplasts, exposure to light induces an excess of D1 synthesis [9,10,11], which provides D1 for PSII repair. Thus, mechanisms must exist to program either stoichiometric or excess D1 synthesis in accord with specific developmental and environmental conditions. Seminal experiments with the green alga *Chlamydomonas reinhardtii* (Chlamydomonas) revealed mechanisms that couple the synthesis of some PSII subunits with their assembly status [12]. However, the underlying mechanisms and the degree to which similar themes apply in plants remain unclear. 

In this study we explored these issues by examining how mutations that disrupt PSII assembly impact the association of ribosomes with chloroplast mRNAs encoding PSII subunits. We focus on mutants lacking four nucleus-encoded proteins: OHP1, OHP2, HCF244, and HCF136. These proteins are conserved in cyanobacteria, where the OHP orthologs are named HliD and HliC, the HCF244 ortholog is named Ycf39, and the HCF136 ortholog is named Ycf48 (reviewed in [3]). Recent work has provided evidence that OHP1, OHP2, and HCF244 (and their cyanobacterial counterparts) comprise a complex required for the addition of nascent D1 to a D2/PsbE/PsbF-containing assembly intermediate [13,14,15,16,17]. Ycf48/HCF136 is believed to act upstream of this step by promoting incorporation of nascent D1 into the OHP1/OHP2/HCF244 complex [14,18,19,20,21]. In addition, there is evidence that these proteins are involved not only in the de novo biogenesis of PSII, but also in PSII repair [14,20]. 

HCF244 was originally discovered in a genetic screen in Arabidopsis [22], at which time it was shown to be essential for PSII accumulation. The PSII defect in the *hcf244* mutant was ascribed to the failure to translate the chloroplast *psbA* mRNA, which encodes D1. Thus, there is evidence that HCF244 functions in both PSII assembly and D1 synthesis, evoking the intriguing possibility that it plays a role in coupling these two processes. The accumulation of HCF244, OHP1, and OHP2 is mutually inter-dependent [14,16], leading to the prediction that *psbA* translation would be similarly affected in *ohp1, ohp2,* and *hcf244* mutants. However, polysome analyses led to the conclusion that OHP1 and OHP2 do not affect the recruitment of ribosomes to *psbA* mRNA [14], contrasting with the polysome data for HCF244 [22].

To clarify the role of the HCF244/OHP1/OHP2 complex in coordinating D1 synthesis with PSII assembly, we used ribosome profiling to analyze chloroplast translation in a maize *hcf244* mutant and in Arabidopsis *ohp1* and *ohp2* mutants. These experiments used deep-sequencing to map and quantify ribosome protected fragments, a method that can be useful for clarifying the interpretation of polysome and pulse-labeling experiments (see, e.g., [23]). Our results show that maize HCF244 is required for *psbA* translation initiation, consistent with the prior conclusions from polysome data in Arabidopsis [22]. However, in contrast with previous conclusions [14], our results indicate that *OHP1* and *OHP2* are likewise required for the recruitment of ribosomes to the *psbA* mRNA. This observation is consistent with the loss of HCF244 when *OHP1* and *OHP2* expression is reduced [14,16]. Importantly, the absence of HCF136, which also functions very early in PSII assembly, does not decrease ribosome occupancy on *psbA* mRNA in maize or Arabidopsis. These results reveal an intimate connection between *psbA* translation and a specific PSII assembly complex, and suggest models for the coupling of D1 synthesis to D1 assembly during PSII biogenesis and repair.

## 2. Results

### 2.1. Maize HCF244 Is Required for the Recruitment of Ribosomes Specifically to psbA mRNA

**Figure 1:**
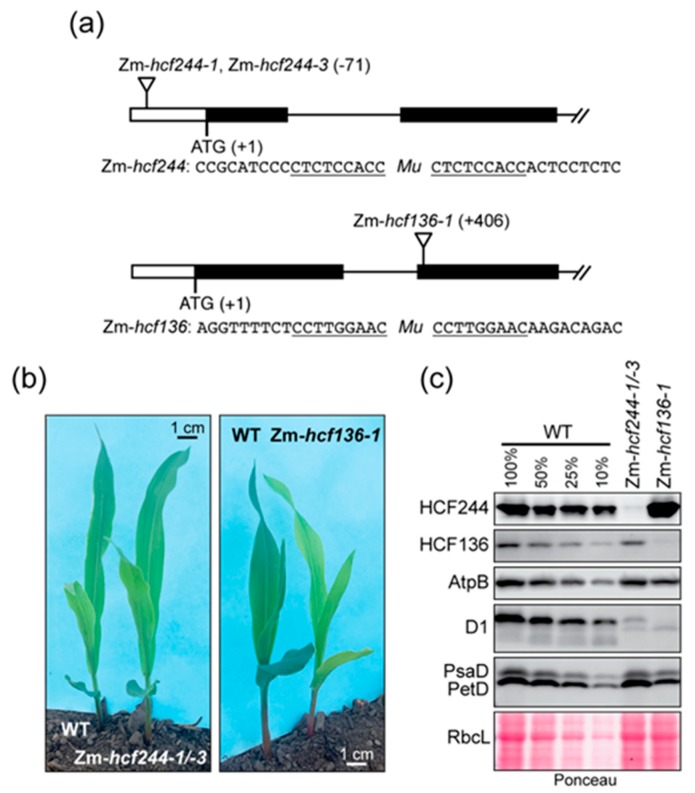
Maize mutants used in this study. (**a**) Insertion sites of *Mu* transposons in Zm-*hcf244* and Zm-*hcf136* mutants. The Zm-*hcf244-1* and Zm-*hcf244-3* alleles arose independently but have an insertion at the same position. The sequences flanking each insertion are shown below, with the target site duplications underlined. (**b**) Mutant seedlings and their phenotypically normal siblings at the developmental stage used for experiments reported here. Plants were grown in soil for eight days as described in Materials and Methods. Zm-*hcf244-1/-3* is the progeny of a cross between heterozygous plants harboring each allele. (**c**) Immunoblot analysis of leaf proteins in Zm-*hcf24*4 and Zm-*hcf136* mutants. Replicate blots were probed to detect HCF244, HCF136, AtpB (subunit of the chloroplast ATP synthase), D1, PsaD (subunit of PSI), and PetD (subunit of the cytochrome *b_6_f* complex). An excerpt of one of the Ponceau S-stained blots illustrates relative sample loading and the abundance of the large subunit of Rubisco (RbcL).

Polysome analysis of an Arabidopsis *hcf244* mutant provided evidence that HCF244 promotes *psbA* ribosome association [22]. However, interpretation of *psbA* polysome data can be challenging due to the large pool of untranslated *psbA* RNA (see, for example [23]). In addition, effects of HCF244 on translation of other chloroplast mRNAs had not been examined. To address these issues, we recovered transposon insertion alleles of maize *hcf244* (Zm-*hcf244*) from the Photosynthetic Mutant Library [24], and analyzed chloroplast translation in the mutants by ribosome profiling. We recovered two independent alleles, which had *Mu* transposon insertions at the identical position in the 5’-untranslated region (Figure 1a). Homozygous mutant seedlings appear very similar to their wild-type siblings when grown in a growth chamber under moderate light conditions, and they grow normally in soil until seed reserves are exhausted (Figure 1b). Immunoblot analysis showed that this insertion conditions a severe loss of HCF244 protein (Figure 1c). As expected based on the *hcf244* mutant phenotype in Arabidopsis, Zm-*hcf244* mutants lack the D1 reaction center protein of PSII (Figure 1c). Core subunits of the chloroplast ATP synthase, Photosystem I (PSI), cytochrome *b_6_f* complex, and Rubisco accumulate normally (Figure 1c), consistent with the established role for HCF244 specifically in PSII biogenesis.

**Figure 2:**
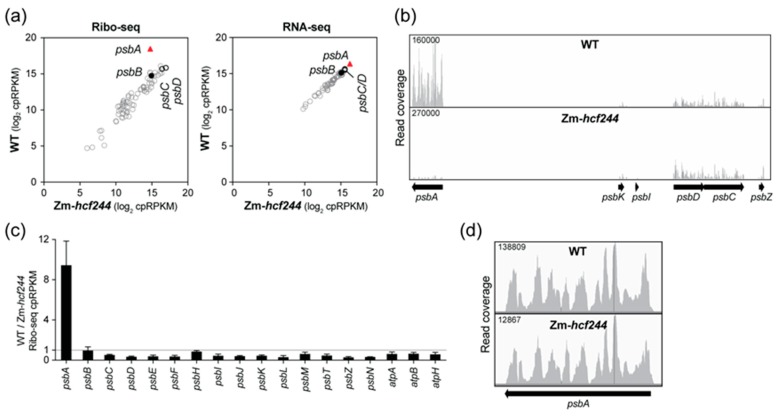
Analysis of chloroplast gene expression in the Zm-*hcf244* mutant by Ribo-seq and RNA-seq. Results from a replicate experiment are shown in Figure S1a. (**a**) Comparison of ribosome footprint abundance (Ribo-seq) and RNA abundance (RNA-seq) for all chloroplast genes in the Zm-*hcf244* mutant and its phenotypically-normal sibling (WT). Each symbol represents one gene. Values are expressed as reads per kilobase in the ORF per million reads mapped to chloroplast ORFs (cpRPKM). (**b**) Screen capture from the Integrated Genome Viewer (IGV) showing the distribution of ribosome footprints along *psbA* and adjacent ORFs in the wild-type and Zm-*hcf244* mutant. The Y-axis shows the number of reads at each position (not normalized); the maximum Y-axis values are shown in the upper left, and were chosen such that peak heights in *psbD* and *psbC* were similar in the two samples. (**c**) Ratio of Ribo-seq reads in the wild-type relative to the mutant for chloroplast genes encoding PSII subunits. Several *atp* genes are shown for comparison. The average of the two replicates (+/– SD) is shown. (**d**) Distribution of ribosome footprints along the *psbA* ORF. The Y-axis maxima were adjusted to facilitate comparison of ribosome distributions in the two genotypes.

We compared chloroplast translation in Zm-*hcf244* mutants to that in their phenotypically normal siblings by sequencing ribosome-protected mRNA fragments (Ribo-seq) recovered from seedling leaf tissue. Two replicate experiments showed a specific loss of ribosomes from *psbA* mRNA in the mutant (Figure 2a,b and Figure S1a). RNA-seq analysis of the same leaf homogenate used for Ribo-seq showed no significant changes in the abundance of chloroplast RNAs (Figure 2a), but slot-blot hybridization analysis suggested a modest (~30%) decrease in *psbA* mRNA abundance in the mutant (Figure S1a). These results indicate that the loss of *psbA* ribosome footprints in the mutant is due almost entirely to reduced translation. To more clearly visualize effects on translation of other chloroplast open reading frames (ORFs) encoding PSII subunits, the relative abundance of ribosome footprints in the wild-type relative to the mutant for these genes is plotted in Figure 2c. This display suggests a slight increase in ribosome footprints on many PSII genes in the mutant; however, this is likely due, at least in part, to the fact that *psbA* ribosome footprints make up such a high fraction of the total footprints in the wild-type; the sharp drop in *psbA* reads in the mutant therefore increases cpRPKM values (calculated by normalizing to total chloroplast reads) for other genes. The residual ribosomes bound to *psbA* mRNA in the mutant were distributed along the ORF similarly to those in the wild-type (Figure 2d), indicating that the loss of ribosomes is not due to ribosome stalling early in the ORF. These results show that HCF244 activates *psbA* translation initiation, validating the conclusion in the original report on Arabidopsis HCF244 [22]. These results show further that HCF244’s function in *psbA* translation is conserved in monocots, and that HCF244 has little or no effect on translation of other chloroplast mRNAs.

### 2.2. OHP1 and OHP2 Are Required to Recruit Ribosomes Specifically to psbA mRNA.

The abundance of HCF244 is strongly reduced in *OHP1* and *OHP2* insertion mutants and knockdown lines [14,16]. Therefore, it might be expected that *ohp1* and *ohp2* mutants would exhibit *psbA* translation defects similar to those in *hcf244* mutants. In fact, pulse-labeling data suggested reduced D1 synthesis in Arabidopsis *ohp1* and *ohp2* mutant and knockdown lines [14,16]. Polysome analyses of *ohp1* and *ohp2* mutants, however, led to the conclusion that OHP1 and OHP2 do not affect *psbA* translation initiation [14]. 

**Figure 3:**
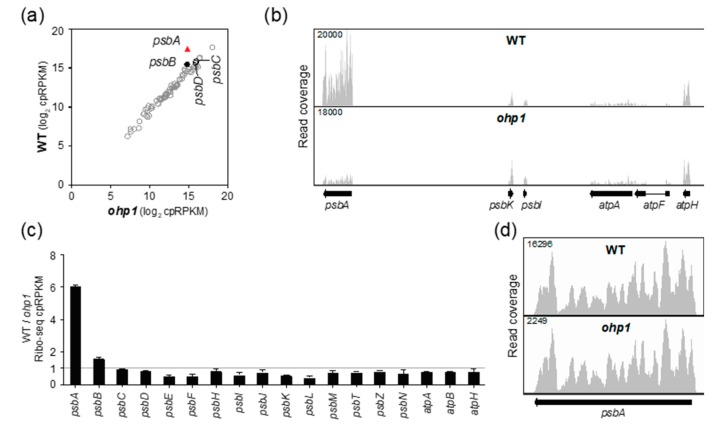
Ribo-seq analysis of Arabidopsis *ohp1* mutants. Data for a replicate experiment are shown in Figure S1b. Symbols and nomenclature are as described in Figure 2. (**a**) Comparison of ribosome footprint abundance for all chloroplast genes in *ohp1* mutants to that in phenotypically-normal siblings. (**b**) Screen capture from the Integrated Genome Viewer (IGV) showing the distribution of ribosome footprints along *psbA* and adjacent ORFs. Different genes are displayed here and in Figure 2b due to the different gene order in the maize and Arabidopsis chloroplast genomes. (**c**) Ratio of Ribo-seq reads in the wild-type relative to the mutant for chloroplast genes encoding PSII subunits. Several *atp* genes are shown for comparison. The average of the two replicates (+/– SD) is shown. (**d**) Distribution of ribosome footprints along the *psbA* ORF. The Y-axis maxima were adjusted to facilitate comparison of ribosome distributions in the two genotypes.

**Figure 4:**
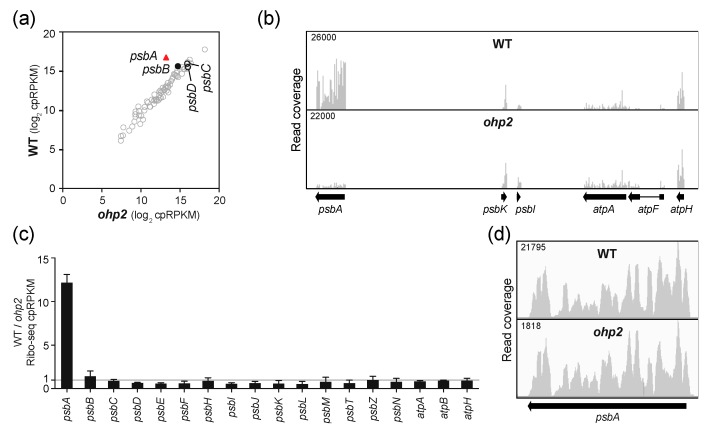
Ribo-seq analysis of Arabidopsis *ohp2* mutants. Data for a replicate experiment are shown in Figure S1c. Symbols and nomenclature are as described in Figure 2. (**a**) Comparison of ribosome footprint abundance for all chloroplast genes in *ohp2* mutants to that in phenotypically-normal siblings. (**b**) Screen capture from the Integrated Genome Viewer (IGV) showing the distribution of ribosome footprints along *psbA* and adjacent ORFs. (**c**) Ratio of Ribo-seq reads in the wild-type relative to the mutant for chloroplast genes encoding PSII subunits. Several *atp* genes are shown for comparison. The average of the two replicates (+/– SD) is shown. (**d**) Distribution of ribosome footprints along the *psbA* ORF. The Y-axis maxima were adjusted to facilitate comparison of ribosome distributions in the two genotypes.

To clarify the role of OHP1 and OHP2 in *psbA* translation, we analyzed Arabidopsis *ohp1* and *ohp2* mutant leaf tissue by Ribo-seq. Both mutants exhibited a strong and specific decrease in *psbA* ribosome footprints (Figure 3a,b and Figure 4a,b). Replicate experiments gave similar results (Figure S1b,c). It was shown previously that *psbA* mRNA accumulates normally in *ohp1* and *ohp2* mutants [14], and we confirmed this to be true by slot-blot or RNA-gel blot hybridization analysis of the same leaf homogenates used for Ribo-seq (Figure S1b,c). The *ohp1* and *ohp2* mutations had little effect on ribosome occupancy on other chloroplast genes encoding PSII subunits (Figure 3c and Figure 4c) and did not significantly alter the distribution of the residual ribosomes on *psbA* mRNA (Figure 3d and Figure 4d). Therefore, OHP1 and OHP2, like HCF244, promote *psbA* translation initiation and do not have strong effects on the recruitment of ribosomes to other chloroplast ORFs. The similar translation phenotypes of the *ohp1, ohp2,* and *hcf244* mutants is consistent with the interdependent accumulation of OHP1, OHP2, and HCF244 [14,16].

### 2.3. Ribosome Occupancy on psbA mRNA Is Not Reduced in hcf136 Mutants 

Results presented above are consistent with the possibility that any defect in assembly of the PSII core reduces *psbA* ribosome recruitment as a secondary effect. However, *hcf107* and *lpe1* mutants, which lack PSII due to defects in expression of the *psbH* and *psbN/psbJ* genes, respectively, show only minor reductions in *psbA* ribosome occupancy [23]. Still, this leaves open the possibility that defects very early in PSII assembly (as in *hcf244*, *ohp1*, and *ohp2* mutants) secondarily cause a defect in *psbA* translation. To address this possibility, we performed similar experiments with mutants lacking HCF136. HCF136 acts upstream of the OHP1/OHP2/HCF244 complex, possibly to facilitate the insertion of D1 into that complex or the insertion of chlorophyll into D1 [14,18,19,20,21]. Furthermore, the HCF136 ortholog in cyanobacteria, Ycf48, associates with the Ycf39/HliD/HliC complex [13], and analysis of *ycf48/ycf39* double mutants suggested that Ycf48 cooperates with the Ycf39 complex to deliver chlorophyll to the assembling PSII reaction center [25]. 

**Figure 5:**
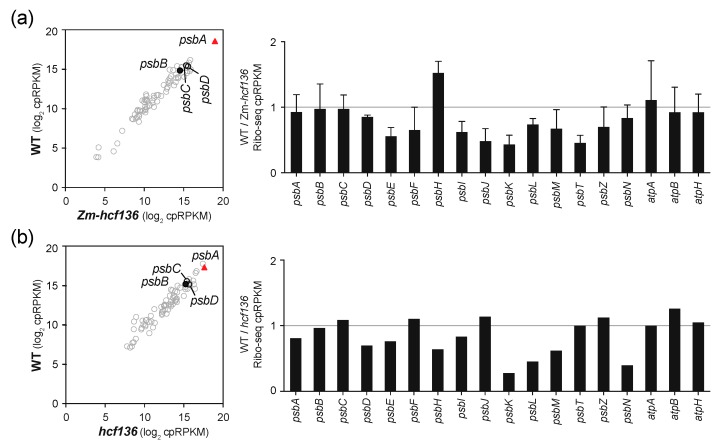
Ribo-seq analysis of *hcf136* mutants in maize and Arabidopsis. Symbols and nomenclature are as described in Figure 2. (**a**) Comparison of ribosome footprint abundance for chloroplast genes in Zm-*hcf136* mutants to that in their phenotypically normal siblings. Results for a biological replicate are shown in Figure S1d. The bar graph shows the average of the two replicates (+/– SD). (**b**) Comparison of ribosome footprint abundance for chloroplast genes in Arabidopsis *hcf136* mutants to that in their phenotypically normal siblings.

To address whether HCF136 impacts the recruitment of ribosomes to *psbA* mRNA, we analyzed *hcf136* mutants in maize (Zm-*hcf136*) and Arabidopsis by Ribo-seq. The Zm-*hcf136* mutant grew similarly to the wild-type and Zm-*hcf244* mutant to the three-leaf stage, but showed a more pronounced chlorophyll deficiency than the Zm-*hcf244* mutant (Figure 1b). D1 abundance is severely reduced in the mutant, whereas core subunits of other photosynthetic complexes were found at near normal levels (Figure 1c). These results are as expected based on the phenotype of Arabidopsis *hcf136* [19] and a previously characterized Zm-*hcf136* allele [26]. Ribo-seq analysis showed no apparent difference in *psbA* ribosome occupancy in the mutant and wild-type in two replicate experiments (Figure 5a and Figure S1d). We obtained similar results with the Arabidopsis *hcf136* allele reported previously [19] (Figure 5b). The *psbA* mRNA was previously shown to accumulate normally in Arabidopsis *hcf136* mutants [19]; we confirmed the same to be true for maize by slot-blot hybridization analysis of RNA from the same extracts used for Ribo-seq (Figure S1d). These results show that an early defect in PSII assembly does not, in and of itself, prevent the recruitment of ribosomes to *psbA* RNA, highlighting the HCF244/OHP1/OHP2 complex as playing a special role in the coordination of PSII assembly with D1 synthesis.

## 3. Discussion

The OHP1/OHP2/HCF244 complex and its ortholog in cyanobacteria facilitate assembly of an early PSII assembly intermediate, and are believed to do so by delivering chlorophyll to nascent D1, scavenging chlorophyll from damaged D1, and/or protecting the assembling reaction center from photooxidative damage [13,14,16,17,27]. HCF244 had, in addition, been reported to promote *psbA* translation initiation [22], whereas polysome analyses of *ohp1* and *ohp2* mutants led to the conclusion that OHP1 and OHP2 do not have this function [14]. These apparently contrasting effects of HCF244 and OHP1/OHP2 on *psbA* translation were puzzling in light of the decreased accumulation of each of these proteins in mutant and knockdown lines for each of the others [14,16]. 

Data presented here resolve this issue by demonstrating that *psbA* translation initiation is strongly and specifically reduced in *ohp1, ohp2,* and Zm-*hcf244* mutants. In fact, inspection of the polysome data reported for *ohp1* and *ohp2* [14] suggests a considerable decrease in ribosome occupancy on *psbA* mRNA. Interpretation of polysome data for *psbA* is challenging due to the unusually small fraction of the mRNA pool that is polysome-associated even in the wild-type. Similarly, inference about rates of D1 and D2 synthesis from pulse-labeling assays in PSII assembly mutants is complicated by their rapid degradation when their assembly is disrupted. These complications in interpreting pulse-labeling and polysome data for *psbA* led to confusion about a role for the pentatricoptide repeat protein LPE1 in *psbA* translation [23,28]. Ribosome profiling or the use of 5’-UTR: reporter gene fusions (e.g., [12]) are better suited to providing unambiguous information about *psbA* translation initiation.

Elegant experiments with Chlamydomonas revealed feedback loops that couple the synthesis of certain chloroplast-encoded subunits of the photosynthetic apparatus with the availability of partner subunits. These phenomena are collectively referred to as Control by Epistasy of Synthesis (CES) [29]. The degree to which CES interactions are conserved in plant chloroplasts remains unclear. For example, CES interactions among ATP synthase subunits that were observed in Chlamydomonas [30] do not occur in maize [31]. Results presented here add to this body of information by addressing whether the D1-dependent translation of the chloroplast *psbB* ORF observed in Chlamydomonas [12] also occurs in plants. Our data show that the severe defect in *psbA* translation initiation in *hcf244, ohp1,* and *ohp2* mutants is not accompanied by a strong decrease in *psbB* ribosome occupancy, suggesting that this CES interaction is not conserved. That said, our data hint at a related phenomenon: The ratio of *psbB* ribosome footprints in the wild-type relative to that in each of these mutants is slightly higher than that for most other chloroplast PSII genes (see Figure 2c, Figure 3c, Figure 4c, and Figure S1); this small effect is not apparent in *hcf136* mutants. We observed a similar weak effect on *psbB* ribosome occupancy in an Arabidopsis *hcf173* mutant, which likewise has a strong defect in *psbA* translation initiation [23,32]. The consistency of this correlation suggests that D1 synthesis mildly enhances PsbB synthesis in plants. It seems possible that this is mechanistically related to the D1-*psbB* CES interaction observed in Chlamydomonas.

**Figure 6:**
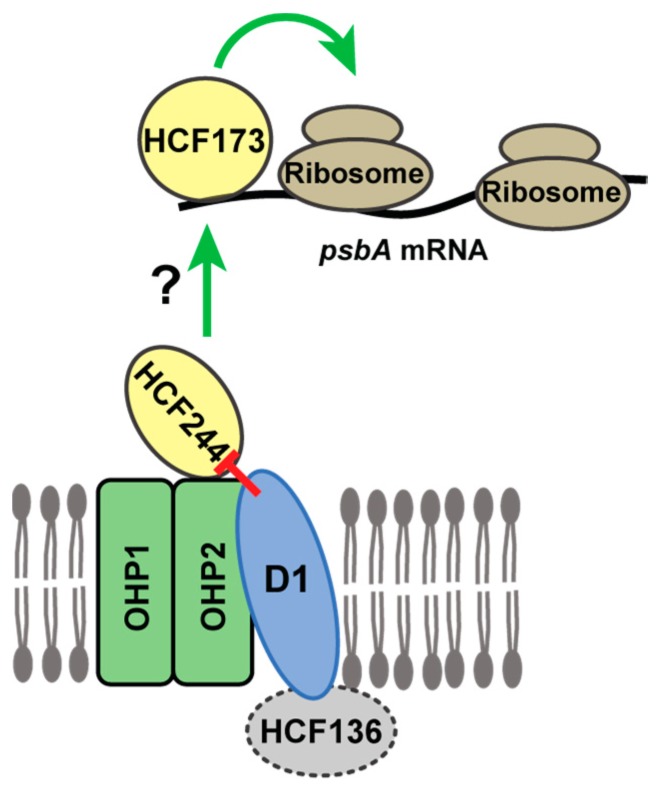
Model for negative autoregulation of *psbA* translation initiation in the context of the HCF244/OHP1/OHP2 complex. OHP1 and OHP2 are integral thylakoid proteins, and HCF244 is tethered to OHP2 on the stromal face of the membrane [13,14,16,17]. HCF173 binds the 5′-UTR of *psbA* mRNA and is required for *psbA* translation initiation [23,32,34]. We propose that stroma-exposed features of the complex (HCF244 and/or OHP2’s stromal tail) collaborate with HCF173 to activate *psbA* translation initiation, and that the presence of D1 in the complex inhibits communication to HCF173. This is a simplified cartoon that does not incorporate evidence that other PSII core subunits (PsbI, D2, PsbE, PsbF) also associate with the HCF244/Ycf39 complex under some conditions [13,14,17]. Ycf48 (the HCF136 homolog in cyanobacteria) associates with this complex when downstream steps in PSII assembly are disrupted, but there is conflicting evidence about the association of HCF136 with the HCF244 complex in plants [13,14,17].

Experiments in Chlamydomonas also revealed that unassembled D1 autoregulates D1 synthesis by repressing *psbA* translation initiation. For example, deletion or truncation of the *psbA* ORF caused a dramatic increase in synthesis of reporter proteins whose expression was driven by the *psbA* 5’ UTR [12,33]. In a similar vein, deletion of all *psbA* genes in *Synechocystis* increased the recovery of ribosomes in Ycf39 affinity purifications [12]. These observations, in conjunction with our results and the recent elucidation of the role of the HCF244/OHP1/OHP2 complex in PSII assembly, suggests that this complex is the hub of an autoregulatory mechanism that couples D1 synthesis with demand for D1. First, the dual effects of the HCF244/OHP1/OHP2 complex on *psbA* translation and PSII assembly are unique to this step in PSII biogenesis: disruption of upstream or downstream steps in PSII biogenesis (e.g., in *hcf136* or *hcf107* mutants) has little or no effect on *psbA* translation initiation (Figure 5 and Reference [23]). Second, there is strong biochemical evidence that nascent D1 is found in a complex together with OHP1/OHP2/HCF244 in plants, and in the corresponding complex in cyanobacteria [13,14,17,27]. That this complex brings D1 into proximity with proteins that are required for the recruitment of ribosomes to *psbA* mRNA suggests a model in which D1 autoregulates *psbA* translation through direct inhibitory interactions with HCF244/OHP1/OHP2 (Figure 6). To elicit effects specifically on *psbA* translation, this complex must influence the activity of one or more proteins that bind directly to *psbA* mRNA. Translation initiates on *psbA* mRNA in the stroma or in loose association with the thylakoid membrane [31]. An exhaustive search for candidate regulators that bind *psbA* RNA in the stroma detected the known translational activator HCF173, but did not detect any other proteins that have strong effects on *psbA* ribosome occupancy [34,35]. As such, we propose that the membrane-associated HCF244 complex communicates in some manner with HCF173 to trigger *psbA* translation (Figure 6). Future experiments will be directed toward testing and elaborating on this model.

## 4. Materials and Methods 

### 4.1. Mutant Lines and Plant Growth. 

OHP1 is encoded by Arabidopsis gene AT5G02120 and OHP2 is encoded by AT1G34000. The *ohp1* and *ohp2* mutants were T-DNA insertion lines GABI_362D02 and GABI_071E10, respectively. These were the same alleles as those used in [36], where they were demonstrated to be null alleles. Both lines were obtained from the Arabidopsis Biological Resource Center. HCF136 is encoded by Arabidopsis gene AT5G23120. The *hcf136* mutant allele was reported in [19] and the seed was a generous gift of Joerg Meurer and Peter Westhoff. Maize HCF136 (Zm-HCF136) is encoded by maize gene GRMZM2G102838 (B73 RefGen v3) or Zm00001d036340 (B73 RefGen v4) and is also called PSB1. Maize HCF244 (Zm-HCF244) is encoded by gene GRMZM2G143917 (B73 RefGen v3) or Zm00001d031997 (B73 RefGen v4). We recovered *Mu* transposon insertion alleles in Zm-*hcf244* and Zm-*hcf136* from the Photosynthetic Mutant Library [24]. The positions of the insertions and evidence that they are null alleles are shown in Figure 1. 

Maize was grown in a growth chamber in diurnal cycles of 16 h light (~200 µE/m^2^/s at 28 °C)/ 8 h dark (26 °C). RNA and ribosome footprints were extracted from the second and third leaves of 7-to-8-day-old seedlings, at which point the third leaf was beginning to emerge. Arabidopsis was germinated and grown on sterile Murashige and Skoog medium (4.33 g/L Murashige and Skoog Basal Medium (Sigma, St. Louis, MO, USA), 2% sucrose, 0.3% Phytagel (Sigma), pH 5.7) in diurnal cycles of 10 h light (80 µE/m^2^/s)/14 h dark at 22 °C.

### 4.2. Ribosome Profiling and RNA-seq.

Ribosome footprint preparation and library construction were performed as described previously [9]. Each replicate experiment performed with the *ohp1* mutant used a pool of three mutant plants. Leaves were harvested at midday on the 18th day post vernalization. The replicate experiments performed with the *ohp2* mutant used pools of four and five mutant plants, respectively. Leaves were harvested at midday on the 15th day post vernalization. The Arabidopsis *hcf136* experiment used a pool of four mutant plants. Leaves were harvested at midday on the 14th day post vernalization. The Zm-*hcf136* replicates and the first replicate of the Zm-*hcf244* experiment used leaves from one mutant seedling, whereas the second Zm-*hcf244* replicate used leaves pooled from two seedlings. Phenotypically normal siblings were used as the wild-type control for all of the experiments except the second replicate of *ohp2* (Figure S1c), for which we used Col-0 grown in parallel.

RNA for RNA-seq was extracted from an aliquot of the same leaf homogenate used for ribosome profiling. Libraries were prepared using Bioo Scientific NEXTflex qRNA-seq kit V2. Data analyses were performed largely as described previously [9], but with the following modifications: (i) Sequence reads were aligned to genomes using STAR instead of Bowtie2; (ii) Read counts excluded the first 10 or 25 nucleotides of each organellar or cytosolic ORF, respectively. Sequencing of Ribo-seq and RNA-seq libraries was performed at the University of Oregon Genomics Core Facility. Read counts for chloroplast genes in Ribo-seq and RNA-seq experiments are summarized in Table S1.

### 4.3. RNA Blot Hybridizations 

RNA gel blot hybridizations were performed as described previously [37]. The *psbA* mRNA was detected on the RNA gel blot with an end-labeled antisense DNA oligonucleotide corresponding to Arabidopsis chloroplast genome (TAIR10) nucleotide positions 1410–1444. Slot-blot hybridizations were performed as described previously [38], using 500 ng of total RNA in each slot. Probes were generated by PCR and radiolabeled with random hexamer priming. The *psbA* probe for both maize and Arabidopsis corresponded to maize chloroplast genome (Accession X86563) nucleotide positions 295–1074. The probe for maize *atpB* probe corresponded to maize chloroplast genome nucleotide positions 54590–55790. The probe for Arabidopsis *atpB* corresponded to Arabidopsis chloroplast genome nucleotide positions 52660–54156. 

### 4.4. Immunoblot Analysis and Antibodies.

Immunoblots were performed as described previously [37]. Proteins were extracted from the apical 2-cm of the second leaf of 8 day old maize seedlings. Antibodies to AtpB, D1, PsaD and PetD were generated by our group and were described previously [39]. Polyclonal antibody to Zm-HCF244 was raised in rabbits (Alpha Diagnostic Intl., San Antonio, TX, USA) using a recombinant antigen corresponding to amino acids 146–385 of Zm-HCF244. The HCF136 antibody was a generous gift of Peter Westhoff and Joerg Meurer.

## Supplementary Materials

The following are available online at https://www.mdpi.com/2223-7747/9/2/152/s1, Figure S1: Supporting data for Ribo-seq assays. Ribo-seq graphs are as described in Figure 2. Slot blots were imaged and quantified with a Storm phosphorimager. (a–c) Replicate Ribo-seq data and *psbA* RNA quantification for Zm-*hcf244, ohp1,* and *ohp2* mutants. The abundance of *psbA* RNA relative to the chloroplast *atpB* mRNA in the extracts used for Ribo-seq was analyzed by slot blot hybridization. The *psbA* RNA in *ohp2* mutants was, in addition, quantified by RNA gel blot hybridization (c, right panel). (d) Replicate Ribo-seq data for Zm-*hcf136* (left panel) and quantification of *psbA* RNA in Zm-*hcf136* extracts used for Ribo-seq (first replicate) (right panel). The abundance of *psbA* RNA relative to *atpB* RNA was analyzed by slot blot hybridization. Table S1: Ribo-seq and RNA-seq read counts for all chloroplast genes.
