# Supplementary material for: Exploring the Link between Photosystem II Assembly and Translation of the Chloroplast psbA mRNA
Source: Plants (Basel). 2020 Jan 25;9(2):152. doi: 10.3390/plants9020152 (PMC7076361; doi:10.3390/plants9020152)
Supplement: Supplementary file 1 [file plants-09-00152-s001.zip › FigS1_v11_Legend.pdf]

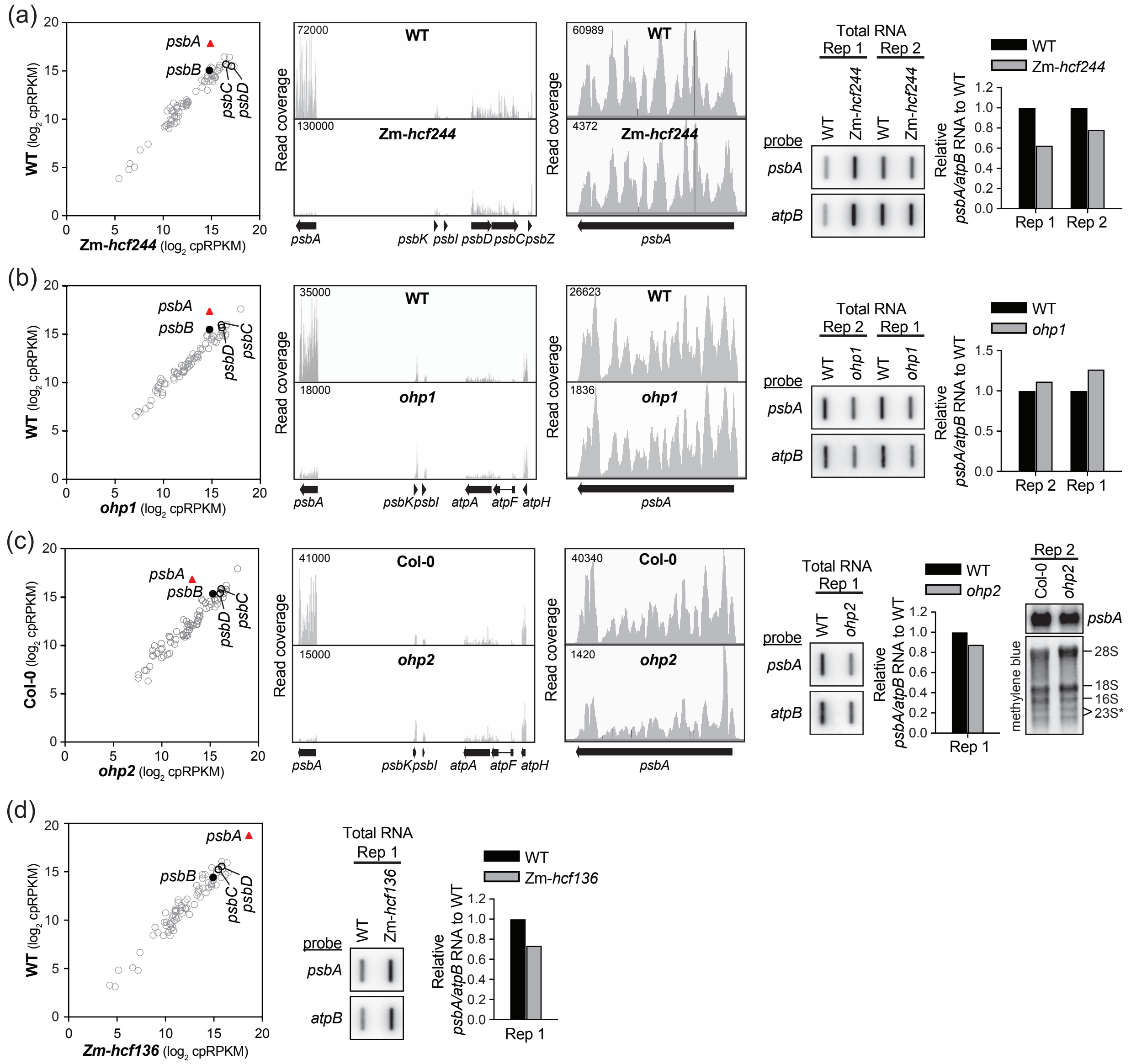

Figure S1: Supporting data for Ribo-seq assays. Plots of Ribo-seq data are as described in **Figure 2**. Slot blots were imaged and quantified with a Storm phosphorimager. **(a-c)** Replicate Ribo-seq data and *psbA* RNA quantification for Zm-*hcf244*, *ohp1*, and *ohp2* mutants. The abundance of *psb*A RNA relative to the chloroplast *atpB* mRNA in the extracts used for Ribo-seq was analyzed by slot blot hybridization. The *psbA* RNA in *ohp2* mutants was, in addition, quantified by RNA gel blot hybridization (**c,** right panel). **(d)** Replicate Ribo-seq data for Zm-*hcf136* (left panel) and quantification of *psbA* RNA in Zm-*hcf136* extracts used for Ribo-seq (first replicate) (right panel). The abundance of *psbA* RNA relative to *atpB* RNA was analyzed by slot blot hybridization.
